# Metric Error Assessment Regarding Geometric 3D Reconstruction of Transparent Surfaces via SfM Enhanced by 2D and 3D Gaussian Splatting

**DOI:** 10.3390/s25144410

**Published:** 2025-07-15

**Authors:** Dario Billi, Gabriella Caroti, Andrea Piemonte

**Affiliations:** ASTRO Laboratory, Department of Civil and Industrial Engineering, University of Pisa, L.go Lucio Lazzarino, 56122 Pisa, Italy; gabriella.caroti@unipi.it (G.C.); andrea.piemonte@unipi.it (A.P.)

**Keywords:** 3D Gaussian splatting, SuGaR, 2D Gaussian splatting, 3D mesh reconstruction, MLP, deep learning, machine learning, NeRFs

## Abstract

This research investigates the metric accuracy of 3D transparent object reconstruction, a task where conventional photogrammetry often fails. The topic is especially relevant in cultural heritage (CH), where accurate digital documentation of glass and transparent artifacts is important. The work proposes a practical methodology using existing tools to verify metric accuracy standards. The study compares three methods, conventional photogrammetry, 3D Gaussian splatting (3DGS), and 2D Gaussian splatting (2DGS), to assess their ability to produce complete and metrically reliable 3D models suitable for measurement and geometric analysis. A transparent glass artifact serves as the case study. Results show that 2DGS captures fine surface and internal details with better geometric consistency than 3DGS and photogrammetry. Although 3DGS offers high visual quality, it introduces surface artifacts that affect metric reliability. Photogrammetry fails to reconstruct the object entirely. The study highlights that visual quality does not ensure geometric accuracy, which is critical for measurement applications. In this work, ground truth comparisons confirm that 2DGS offers the best trade-off between accuracy and appearance, despite higher computational demands. These findings suggest extending the experimentation to other sets of images featuring transparent objects, and possibly also reflective ones.

## 1. Introduction

The metric accuracy validation and reconstruction of three-dimensional geometries from input data is a fundamental challenge in remote sensing and related fields. This process is often both time-intensive and complex, particularly when dealing with existing structures and the intricate nature of building stocks. These challenges are further exacerbated in the context of CH, where structures are often characterized by irregular geometries, heterogeneous materials, and unique aesthetic and historical values [1]. It is important to note that CH includes not only historical artifacts but also modern cultural assets, such as objects of industrial design and contemporary material culture, which also require accurate digital preservation and documentation.

Photogrammetric approaches, such as structure from motion and multi-view stereo (SfM-MVS), are frequently employed for 3D reconstruction [2]. However, these methods often fall short in capturing critical surface details, especially in cases involving transparent materials [3,4]. These shortcomings highlight the limitations of conventional techniques when applied to complex artifacts or scenes, whether historical or modern. In particular, objects made of glossy or multi-material transparent components, such as glass sculptures, have proven especially challenging to digitize using photogrammetry alone, often requiring hybrid solutions for example combining photographic and tomographic methods to achieve satisfactory results [5].

Recent advances in technology, particularly the integration of computer vision (CV) and artificial intelligence (AI) into photogrammetry, have opened up new possibilities for overcoming these challenges. Emerging methodologies, including neural radiance fields [6] and 3DGS [7], offer promising pathways for enhancing the accuracy and efficiency of 3D reconstructions. These techniques aim to address the limitations of traditional methods by leveraging data-driven models that adapt to complex and variable surface properties.

Related to these methodologies, the literature offers numerous studies assessing the visual quality of reconstructions often relying on rendering metrics such as peak signal-to-noise ratio (PSNR), structural similarity index measure (SSIM) [8], and learned perceptual image patch similarity (LPIPS) [9]. A visually plausible and correct reconstruction according to CV metrics is not sufficient to create the digital twin of the surveyed object; it is therefore essential for this purpose that the reconstructed surfaces are also validated using accuracy and geometric fidelity indexes, as demonstrated in benchmark studies on surface reconstruction assessment in photogrammetric applications [10] and in practical case studies such as Quality Evaluation of Digital Twins Generated Based on UAV Photogrammetry and TLS: Bridge Case Study [11].

Papers on CV already employ a sophisticated set of metrics to assess the quality of the results, and many of these metrics indirectly reflect the geometric quality of the model; however, our approach focuses on an explicit and systematic analysis of the geometric component.

For our purposes, it is crucial to have tools and data that allow us to evaluate the geometric accuracy of the digital twin model in a precise way.

In this work we present a comparative methodology based on a controlled workflow, involving the acquisition of RAW images in a neutral environment and the processing of a test dataset: a transparent glass bottle, representative of contemporary transparent objects with cultural and symbolic value. The process is structured into three parallel pipelines, all deriving from a common sparse point cloud generated in SfM. The pipelines differ in the reconstruction technology employed:

- SfM, photogrammetric approach
- 3DGS (tridimensional Gaussian splatting)
- 2DGS (bidimensional Gaussian splatting)

All resulting models are evaluated both quantitatively (through metric comparison with a reference ground truth model) and qualitatively (through visual comparison and rendering metrics). The results provide an objective assessment of the potential and limitations of each approach, highlighting the effectiveness of AI-based techniques in improving the reconstruction of challenging transparent surfaces in applications that encompass modern and industrial cultural assets.

## 2. State of Art

The field of 3D reconstruction has undergone significant transformation with the advent of AI and CV techniques [12,13,14]. SfM-MVS has significantly simplified surveying workflows and accelerated post-processing. While these methods are highly effective for many applications, their limitations in handling complex materials and geometries have been well-documented. For instance, reflective, transparent, or homogeneous surfaces often lead to data gaps or inaccuracies in standard workflows.

In recent years, researchers have increasingly turned to advanced algorithms and machine learning models to address these challenges. Neural radiance fields (NeRFs) [6,15] have emerged as a promising technology for photorealistic 3D reconstruction, particularly for scenes with intricate lighting and material interactions [16]. NeRFs model the radiance emitted from a 3D scene using deep neural networks, allowing for the synthesis of novel views.

Similarly, 3DGS [7] has gained attention for its ability to efficiently approximate complex surface geometries using a probabilistic framework. By representing scenes as a collection of Gaussian splats, this method reduces computational overhead while maintaining accuracy in rendering, making it suitable for real-time applications.

These advancements reflect a broader trend towards hybrid methodologies that combine SfM-MVS with AI-driven solutions. Such approaches not only improve the metric accuracy of reconstructions but also enhance their visual realism, offering significant benefits for applications in CH and beyond.

### 2.1. Optimizations from the Original Paper of 3DGS

The Gaussian splatting technique represents a particularly promising direction for 3D reconstruction, as evidenced by the growing interest within the scientific community and the increasing number of publications dedicated to the subject. Among the most recent and significant contributions reviewed for this work, particular attention is given to implementations of Gaussian splatting aimed at generating solid meshes from the set of Gaussians, as well as to recent strategies focused on improving and optimizing the reconstruction of transparent objects.

#### 2.1.1. Surface Mesh Extraction

Surface mesh extraction is a crucial task in computer graphics and CV, aimed at generating a 3D mesh from various representations of objects or scenes. Mesh-based representations are essential for editing, sculpting, animating, and relighting.

SuGaR [17]: Introduces a regularization term that aligns Gaussians with the scene’s surface, enabling mesh extraction via Poisson reconstruction. This method is fast, scalable, and preserves detail, unlike the Marching Cubes algorithm, typically used for mesh extraction from neural SDFs. SuGaR also offers an optional refinement strategy that binds Gaussians to the mesh surface, jointly optimizing both the Gaussians and the mesh through Gaussian splatting rendering.

GS2Mesh: Gaussian splatting-to-mesh [18] bridges the gap between noisy 3DGS and a smooth 3D mesh by incorporating real-world knowledge into the depth extraction process. Instead of directly extracting geometry from Gaussian properties, GS2Mesh uses a pre-trained stereo-matching model to guide the process. It renders stereo-aligned image pairs, feeds them into a stereo model to obtain depth profiles, and fuses these profiles into a single mesh. This approach results in smoother, more accurate reconstructions with finer details compared to other surface reconstruction methods, with minimal overhead on top of the 3DGS optimization process.

GOF: Gaussian opacity fields [19] proposes an efficient and high-quality method for surface reconstruction in unbounded scenes. Building on ray-tracing-based volume rendering of 3D Gaussians, GOF directly extracts geometry by identifying the level set of Gaussians, bypassing Poisson reconstruction or TSDF fusion. It approximates surface normals through the ray–Gaussian intersection plane and applies regularization to improve geometry accuracy. Additionally, GOF introduces an efficient geometry extraction technique using marching tetrahedra, adapting to scene complexity.

2DGS: 2D Gaussian splatting [20] introduces a novel approach by collapsing 3D volumes into 2D oriented planar Gaussian disks. Unlike 3D Gaussians, 2D Gaussians model surfaces consistently from different views. 2DGS employs a perspective-accurate splatting process with ray–splat intersections and rasterization to recover thin surfaces accurately. It also integrates depth distortion and normal consistency terms to enhance reconstruction quality.

2DGS simplifies the 3D volume by representing it as a collection of 2D oriented planar Gaussian disks, which ensure view-consistent geometry [20]. To improve the quality of reconstructions, two regularization terms are introduced: depth distortion and normal consistency. The depth distortion term focuses on concentrating the 2D primitives within a narrow range along the viewing ray, while the normal consistency term ensures that the rendered normal map aligns with the gradient of the rendered depth.

MVG-Splatting: Multi-view guided Gaussian splatting with adaptive quantile-based geometric consistency densification [21] is a method that utilizes depth-normal mutual optimization to guide a more precise densification process, enhancing the detail representation for scene rendering and surface extraction. Building on the 2DGS framework [20], it implements a more robust technique for recalculating surface normals. These recalculated normals, combined with gradients from the original images, help refine the accuracy of the rendered depth maps. It then proposes an efficient multi-level densification approach based on multi-view geometric consistency [22], which directs the refined depth maps to accurately project onto under-reconstructed regions. In contrast to previous GS-based geometric reconstruction methods [18,23,24,25], its approach first generates high-quality, uniformly densified Gaussian point clouds. This allows for direct surface extraction using the Marching Cubes method [26] on the point cloud. Additionally, it adaptively determines voxel sizes for each densified Gaussian point cloud and uses multi-view normal maps to smooth and optimize surface normals, resulting in high-detail mesh surface extraction.

The MeshSplats [27] method addresses the limitation of 3DGS (that typically bypasses classical methods such as ray tracing, thereby missing out on key benefits like handling incoherent rays essential for complex lighting effects such as shadows and reflections), by converting the Gaussian elements generated into a mesh-like representation, enabling direct use of ray tracing. This approach allows for an immediately usable mesh following the conversion, albeit with slightly reduced quality compared to the original model. However, reconstruction quality can be further enhanced through a dedicated optimization algorithm that operates directly on mesh faces rather than on Gaussian components. Experimental results demonstrate the effectiveness of the method and its potential applications in computer graphics and image processing.

#### 2.1.2. New Enhancement and Optimization of Transparent Object Reconstruction

Recent advancements have addressed the specific challenges posed by transparent objects in 3D reconstruction, leveraging Gaussian splatting frameworks enhanced with priors and embedding techniques to improve surface accuracy and visual fidelity.

At the time this research was conducted, several of the most recent approaches despite their high potential and relevance had not yet been formally published or had not released open-source implementations available for testing and validation by the scientific community. As Gaussian splatting and its applications to transparent object reconstruction remain a rapidly evolving field, these methods could not be included in the experimental evaluation. Future research will aim to integrate and benchmark these emerging techniques as their implementations become publicly accessible, with the objective of further advancing the precision, reliability, and applicability of 3D reconstruction in complex transparent scenarios.

TSGS: Improving Gaussian splatting for transparent surface reconstruction via normal and de-lighting priors [28] introduces a novel approach that integrates normal vector priors and de-lighting constraints to better handle the complex light interactions typical of transparent surfaces. This method refines the Gaussian splatting representation by correcting lighting distortions and enhancing surface normal consistency, resulting in improved geometric accuracy and visually coherent reconstructions. TSGS also implements an optimization strategy that adjusts splat parameters in response to the learned priors, reducing artifacts commonly found in transparent object reconstructions.

TranSplat: Surface embedding-guided 3D Gaussian splatting for transparent object manipulation [29] proposes an innovative framework that incorporates surface embedding techniques to guide the Gaussian splatting process for transparent objects. By embedding surface features learned from multi-view images, TranSplat achieves more precise modeling of thin and layered transparent structures, enabling not only accurate reconstruction but also interactive manipulation of transparent surfaces. This approach enhances detail preservation and improves the stability of reconstructions under varying lighting and viewing conditions, making it particularly suitable for applications in cultural heritage digitization and virtual display.

These methods represent a significant step forward in the metric and visual quality of transparent object reconstruction, overcoming many limitations of traditional photogrammetry and earlier Gaussian splatting implementations. They showcase the potential of combining prior knowledge and embedding strategies to address the unique challenges of transparent materials, supporting more reliable and detailed 3D models essential for precise measurement and analysis.

The GlassGaussian method [30] extends 3DGS by modeling realistic imperfections and glass materials. This method addresses challenges specific to transparent surfaces by integrating diffraction and refraction models into the Gaussian splatting framework, producing highly realistic and geometrically faithful reconstructions. GlassGaussian is especially valuable for digital preservation in cultural heritage and realistic rendering of artistic glass objects.

### 2.2. Metrics

#### 2.2.1. Rendering Metrics

In standard settings for novel view synthesis using 3DGS, visual quality assessment metrics are used for benchmarking. The most widely adopted metrics in the literature include peak signal-to-noise ratio (PSNR), structural similarity index measure (SSIM) [8], and learned perceptual image patch similarity (LPIPS) [9].

PSNR is used to compare the similarity between rendered images generated by models and real images. A higher PSNR value indicates greater similarity and better image quality. However, PSNR has some limitations as it primarily focuses on mean square error, overlooking human eye sensitivity to different frequency components and the effects of perceptual distortions. As a result, PSNR may not always accurately reflect the perceived differences in image quality from a human perspective.

SSIM is a metric for measuring the structural similarity between two images, taking into account brightness, contrast, and structure. SSIM values range from −1 to 1, with values closer to 1 indicating higher similarity between the two images. 

LPIPS evaluates the similarity between two images based on feature representations extracted from a pre-trained deep neural network. The original LPIPS paper used SqueezeNet [31], VGG [32], and AlexNet [33] as feature extraction backbones. LPIPS scores are more closely aligned with human perceptual judgments compared to traditional metrics like PSNR and SSIM. A lower LPIPS score indicates higher similarity between the images.

Unlike traditional metrics such as PSNR and SSIM, which calculate differences based on raw pixel values or simple transformations thereof, LPIPS leverages deep learning to better align with human visual perception. It uses the distance between features extracted by a convolutional neural network (CNN) pretrained on an image classification task as a perceptual metric.

#### 2.2.2. Geometric Metrics in 3D Surveying

In the evaluation of geometric quality for 3D reconstructions, particularly in topographic surveys and photogrammetry, it is important to employ quantitative metrics that comprehensively describe the fidelity of the reconstructed model with respect to the reference ground truth. Among the most widely used and recognized metrics in the scientific literature [34,35,36,37,38,39,40], assuming the errors follow a normal distribution, are five key indexes: the mean error, the standard deviation of the error, the number of points within a tolerance range, the root mean square error (RMSE), and the completeness.

The mean represents the average point-wise deviation between the reconstructed model and the ground truth. This index allows for assessing the presence of systematic bias in the reconstruction, with values close to zero indicating negligible average error.

The standard deviation quantifies the dispersion of errors around the mean, highlighting the consistency and stability of the reconstruction process. A low standard deviation indicates a uniform and controlled reconstruction, whereas higher values may point to regions with greater errors or measurement noise.

The points within tolerance range counts the number of points in the reconstructed cloud whose distance from the reference model is below a predefined threshold. This threshold represents the maximum error considered negligible or acceptable meaning differences within this limit are not treated as significant errors. This metric defines which points are deemed accurate and directly impacts the completeness of the reconstruction.

The RMSE [41] is a statistical measure that quantifies the difference between values predicted by a model and the values actually observed. It represents the square root of the average of the squared errors made. In other words, the RMSE is calculated by considering both the square of the mean, also called bias, and the square of the standard deviation, which represents the random dispersion of errors. The RMSE is therefore the square root of the sum of these two values. A lower RMSE value indicates higher accuracy and a better ability of the model to reproduce the observed data.

Completeness can be defined as the percentage of reference model points accurately reconstructed within the tolerance threshold. It is calculated as the ratio between points within range and the total number of points considered (including out-of-range points).

## 3. Materials

The pilot case study consists of a glass bottle (Mario Luca Giusti transparent bona bottle, manufactured by Mario Luca Giusti S.r.l., based in Serravalle, Italy) placed on a motorized turntable against a uniform neutral-gray background.

**Figure 1:**
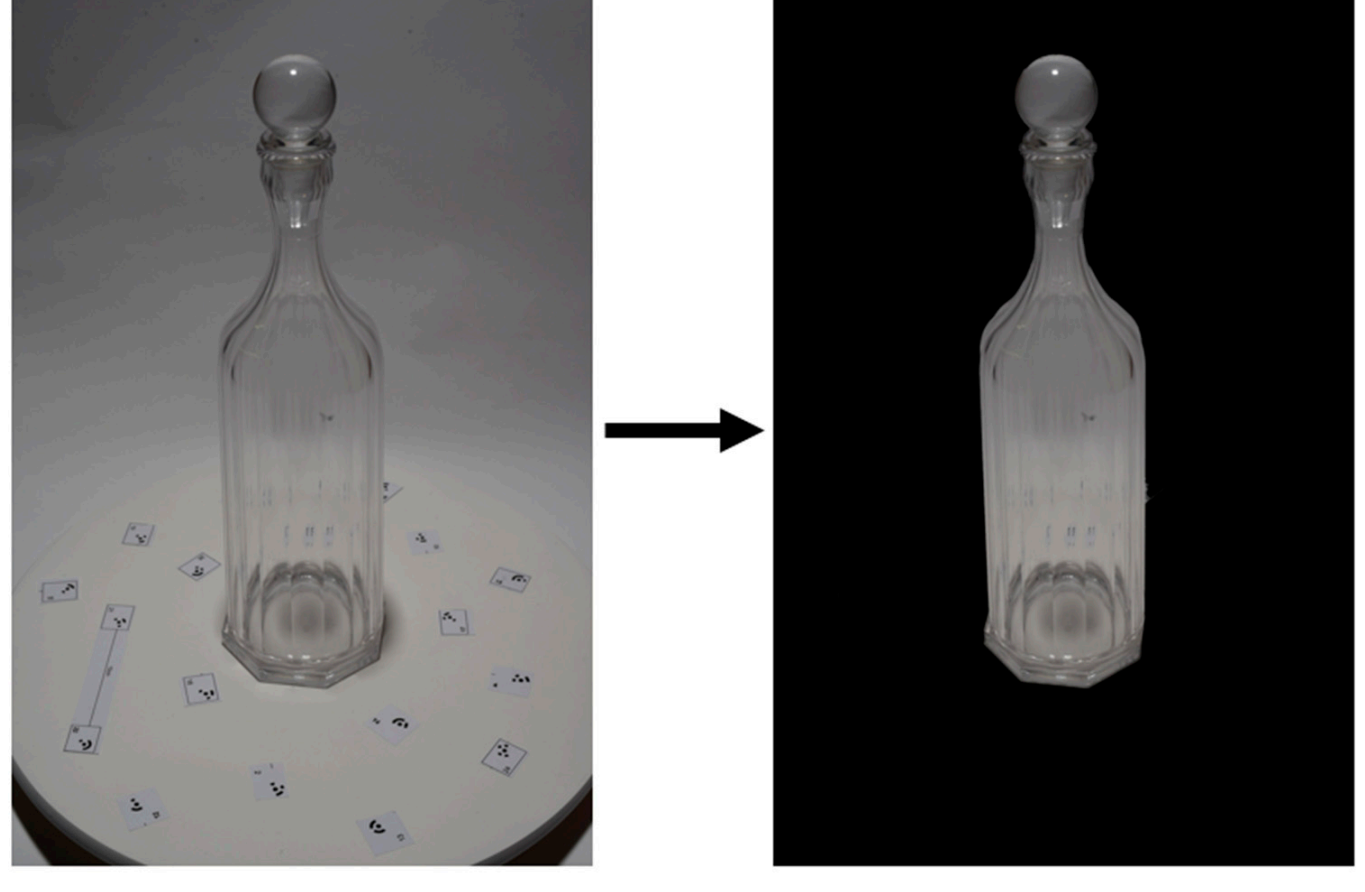
Representative image pair: the left panel displays the original RAW capture, while the right panel demonstrates the result after automated masking.

The images were acquired in a controlled environment and subsequently processed using Adobe Lightroom Classic v14.0.1 (Figure 1), employing the automatic masking system based on contour recognition to isolate the object of interest. The mask was then manually refined in areas with soft transitions or poorly defined edges to achieve more precise selection.

It is important to underline that masking was not applied during the initial photogrammetric alignment phase, in order to avoid interfering with the detection of markers. These markers consist of coded targets that are automatically recognized by the photogrammetric software. In particular, the double markers with integrated scale bars serve not only to define the scale of the model but also to provide fixed reference distances, thereby improving the geometric accuracy of the reconstruction. The use of such coded markers is supported in the literature as a method that reduces collimation error compared to manual point selection [42]. Automatic detection ensures greater consistency and precision, minimizing human error in marker identification and thereby contributing to a more accurate scaling and alignment of the final 3D model. During the alignment phase, the software also detects points in background areas or reflective surfaces, which can introduce noise but may still provide useful information for the reconstruction. For this reason, these elements were not filtered out at this stage.

Masking was therefore applied after the alignment phase, with the aim of optimizing the images used in the different reconstruction workflows. In generic SfM software, masking helps reduce the influence of distracting elements such as the background or reflections during the generation of the dense point cloud and mesh [43]. In the case of Gaussian splatting software, masked images allow for the exclusion of irrelevant areas like backgrounds and reflections during the training and Gaussian optimization stages, thus contributing to higher quality and consistency in the final model [44].

Based on the experimental observations collected in this study, background removal led to cleaner and more accurate models, with reduced noise and greater uniformity in reconstruction. Throughout the entire process, the images were preserved in their original RAW format, with no alteration to resolution or metadata.

To ensure model scalability and metric error control, the photogrammetric survey was supported by a high-precision topographic survey. During this topographic survey, the coordinates of 27 markers placed on the rotating platform were acquired using a total station Leica TCRP 1201 (manufactured by Leica Geosystems AG, Heerbrugg, Switzerland), with an accuracy of up to one-tenth of a millimeter.

The 227 images of the pilot dataset were acquired with a Nikon D750 camera (manufactured by Nikon Corporation, Tokyo, Japan). The camera’s characteristics and common image acquisition settings are reported in Table 1.

Finally, to create a reference model (ground truth) for comparing different processing software, an initial acquisition was carried out using a glass bottle entirely covered with a paper sticker (of negligible thickness), which was then hand-painted in order to create a variegate pattern for the texture. This setup enabled a complete photogrammetric reconstruction, avoiding issues related to transparency or unwanted reflections. The ground truth was acquired using the same camera settings detailed in Materials section, and the alignment and mesh-creation parameters were identical to those reported in Methodology section.

In this work, several processing tools rely on command-line execution, requiring the use of scripts to automate and coordinate the workflow. To facilitate the execution of these scripts and ensure a consistent software environment, we used Anaconda (v.conda 23.7.4), a widely adopted platform that manages packages and environments for Python v.3.7.13 and other languages. This setup enables the proper functioning of the GS-based methodologies and related processing pipelines, which are primarily launched and controlled via the command line. Tests were conducted using an NVIDIA GeForce RTX 4090 GPU (24 GB VRAM) (Nvidia, Santa Clara, CA, USA) and an AMD Ryzen 97950X 16-Core CPU (AMD, Santa Clara, CA, USA).

## 4. Methodology

The methodology adopted in this study was designed to compare and extract the metrics at the Section 2.2. of different 3D reconstruction technologies, starting from a common input. The goal is to obtain a comparative evaluation of the results generated by each process, both quantitatively (through objective metrics) and qualitatively (through visual analysis).

### 4.1. Common Input and Initial Phase

The methodology begins with the definition of a common input, which consists of the internal and external orientation parameters of the cameras. These parameters are computed within a single SfM environment and represent the solution to the photogrammetric problem. Based on this common input, it becomes possible to generate a sparse point cloud, which serves as an initial geometric representation of the scene and forms the basis for subsequent processing steps. This operation is performed using the open-source software COLMAP v. 3.12.1, which is widely recognized for its effectiveness in 3D reconstruction based on SfM. Nonetheless, COLMAP struggled to align all the images correctly in our dataset, leading to incomplete reconstructions and inconsistencies in camera parameter estimation. For this reason, we opted to use Agisoft Metashape (AM) v.2.0.0, a different SfM software, that ensured a complete and coherent dataset’s image alignment. Since the 3DGS and 2DGS methods necessarily require input files in the COLMAP format, an export script was used to generate compatible files from the alignment parameters computed in AM, without altering their values, but simply reorganizing them according to the COLMAP format structure.

The common parameters for the alignment are shown in Table 2.

The implemented workflow began with the determination of the internal and external orientation parameters of the cameras, using the raw unmasked images with automatically detected markers. Subsequently, a systematic path replacement was carried out using the “Change Path” command, in order to switch to the masked image set while carefully preserving the previously established camera alignment parameters.

### 4.2. Parallel Reconstruction Processes

**Figure 2:**
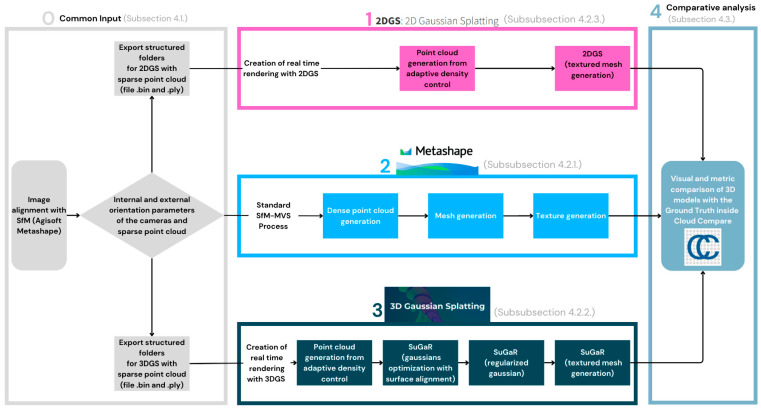
Overview of the proposed methodology.

The methodology involves the use of three distinct approaches for 3D reconstruction, each based on different technologies and software (Figure 2):

#### 4.2.1. SfM Agisoft Metashape (AM) v.2.0.0

The first process is based on the SfM technique, which uses photogrammetric algorithms to reconstruct 3D geometry from 2D images. The parameters for generating the model are shown in Table 3.

Surface Type set to Arbitrary, Interpolation enabled, and Depth Filtering set to Mild are simply the defaults for the Depth Maps workflow and do not alter the reconstruction beyond the standard configuration.

#### 4.2.2. 3D Gaussian Splatting (3DGS) Latest Code Update on August 2024 + SuGaR Latest Code Update on September 2024

The 3DGS technique uses 3D Gaussians to represent the scene. A Gaussian in this context is a density distribution used to model the light and energy radiated from a point in space. However, 3DGS has limitations: 3DGS does not natively support mesh extraction in its official base code. To overcome this issue, we chose to adopt SuGaR, (one of the implementations mentioned in Section 2.1.1), which extends 3DGS and is specifically designed to enhance surface mesh reconstruction. SuGaR combines Gaussian-based representation with enhanced surface awareness, enabling more precise and detailed reconstruction of complex geometries. Specifically, SuGaR introduces an optimization process that aligns 3D Gaussians with the actual surfaces of objects, reducing distortions and improving consistency across different viewpoints.

Thanks to SuGaR, it is possible to obtain more accurate meshes that adhere closely to real surfaces, solving many of the issues related to the representation of thin edges and fine details. This approach is particularly useful in applications such as 3D reconstruction from images, virtual reality, and augmented reality, where surface precision is critical.

Below are the scripts executed in the Anaconda prompt for the generation of the 3DGS model and the creation of the mesh model using SuGaR, with all other parameters left at their default values:

- python train.py -s data/bottle2025mask -r 2 --iterations 30,000 [3DGS]
- python train.py -s data\bottle2025mask -c gaussian_splatting\output\bottle2025mask\ -r dn_consistency --refinement_time long --high_poly True -i 30,000 [SuGaR]

#### 4.2.3. 2D Gaussian Splatting (2DGS) Latest Code Update on December 2024

The 2DGS approach overcomes the limitation of 3DGS by representing the scene as a set of 2D Gaussians. Instead of using Gaussians distributed in 3D space, the Gaussians are projected onto oriented planes (like disks) that describe the surfaces of the scene. This method is advantageous because 2D Gaussians are more view-consistent, ensuring more accurate and stable geometry across different views of the scene.

To achieve accurate reconstruction, 2DGS introduces a 2D splatting process (an operation that projects light from 2D points onto the 3D scene) that accounts for perspective correctness and ray–splat intersections. Furthermore, the process uses rasterization (a method for “drawing” images on a grid) to achieve detailed visual rendering.

Additionally, two optimization terms are employed:

- Depth distortion: Corrects errors in the perceived depth between objects.
- Normal consistency: Ensures consistency in surface normals (the direction of surface planes) to maintain coherent surface representation across views.

The main advantage of 2DGS is that it enables stable and detailed geometric reconstruction of surfaces without visible noise. Moreover, it maintains high visual quality, ensures fast training speeds, and allows real-time rendering [20]. This makes it suitable for applications requiring high-quality visualization and real-time performance.

The 3DGS method evaluates scene values using different intersection planes depending on the viewpoint from which the scene is observed. This approach can lead to inconsistencies, as the representation of geometry or surfaces may vary slightly when the scene is viewed from different angles. For example, thin surfaces or edges might appear distorted or imprecise depending on the perspective.

In contrast, the proposed 2DGS method solves this problem by providing consistent multi-view evaluations. Instead of using variable intersection planes, 2DGS represents the scene through oriented 2D Gaussian disks, maintaining a uniform and consistent representation regardless of the viewing angle [20]. This ensures greater accuracy in surface reconstruction and better visual coherence, especially in applications such as novel view synthesis or 3D reconstruction.

**Figure 3:**
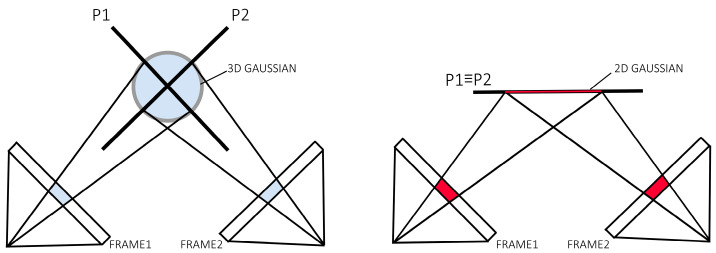
Comparison of 3DGS and 2DGS. 3DGS utilizes different intersection planes P1 and P2 for value evaluation when viewing from different viewpoints, resulting in inconsistency. 2DGS provides multi-view consistent value evaluations.

In summary, while 3DGS can suffer from inconsistencies due to its viewpoint dependency, 2DGS offers a more robust and reliable solution, maintaining a consistent and precise representation from any angle (Figure 3).

Below are the scripts executed in the Anaconda prompt for the generation of the 2DGS mesh model, with all other parameters left at their default values:

- python train.py -s data/bottle2025mask -r 2 -iterations 30,000 [2DGS]
- python render.py -m output\bottle2025mask -s data\bottle2025mask [2DGS]

Among the various extensions of Gaussian splatting, SuGaR and 2DGS were selected because, in addition to having available and actively maintained source code for both Linux and Windows platforms, they are among the more established and commonly used implementations within the GitHub user community (available at the link https://github.com/). We tested different numbers of training iterations and observed negligible variations in geometric accuracy (our main focus). As a result, we adopted the default setting of 30 k iterations for both 3DGS and 2DGS processes. Training time assessments were based on this configuration.

### 4.3. Comparative Analysis

Once the three reconstruction processes are completed, the results undergo a comprehensive comparative analysis against the ground truth. This comparison involves evaluating quantitative metrics, such as accuracy and precision, through distance measurements between models, as well as qualitative assessments based on visual inspection of the reconstructed outputs. Although this comparison can be performed using various software tools, the open-source software CloudCompare v.2.13.2 was selected for its user-friendly built-in features, including the cloud-to-mesh (C2M) distance computation, which enables precise evaluation of differences between reconstructed models and the ground truth mesh. This phase aims to assess each method’s performance in terms of reconstruction quality, processing time, and adaptability to different scene types.

## 5. Results and Comparative Analysis

The results obtained through the comparison of the various outputs from parallel processes using CloudCompare are divided into a qualitative analysis of the generated mesh models and a subsequent metric analysis for a more detailed evaluation of the differences. Although the primary focus is on metric evaluation, qualitative analysis remains an important step for visually verifying the reconstruction quality.

### 5.1. Qualitative Analysis

The analysis was conducted by displaying different views (lateral, top, and perspective) of the reconstructed models. Additionally, the mesh models were visualized without textures (shaded models). This is useful for evaluating the quality of the reconstruction from various angles, avoiding potential artifacts introduced by textures that could mislead the geometric visualization of the mesh.

**Figure 4:**
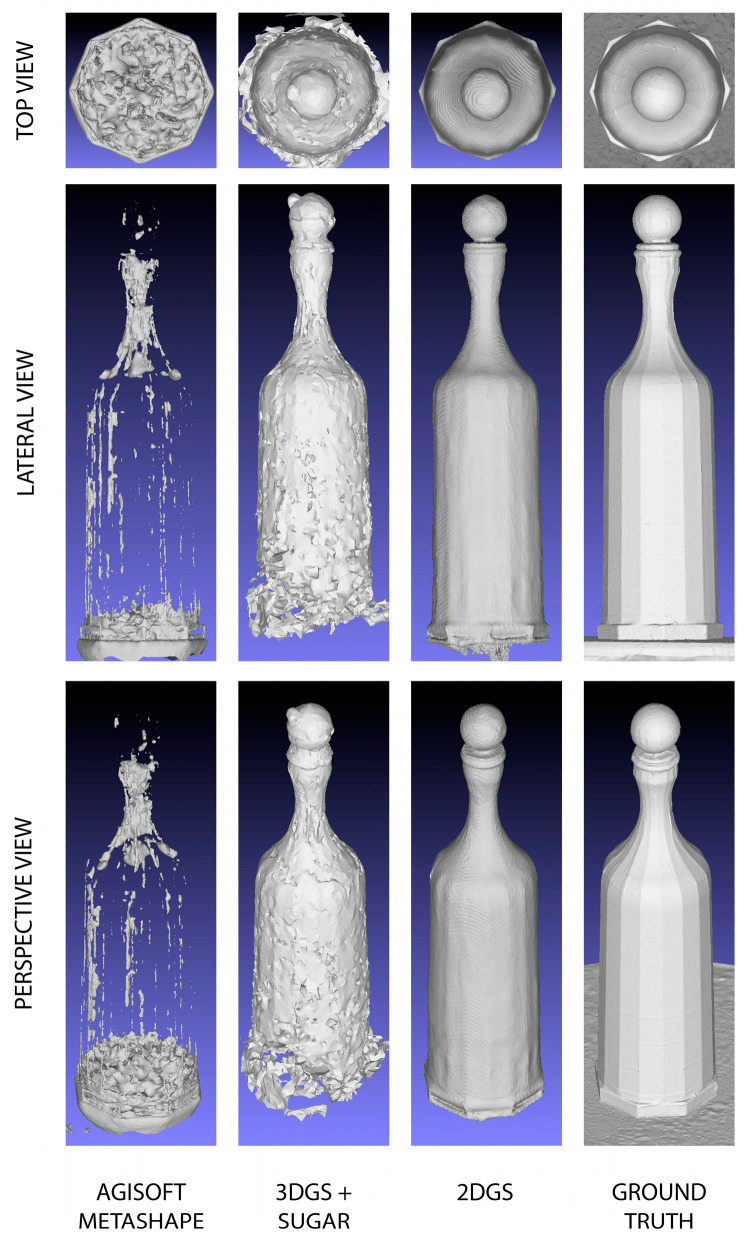
Visual comparisons of the meshes reconstructed by the three different processes.

As can be seen from the Figure 4, the process using AM completely fails in reconstructing the mesh model. The process based on 3DGS with SuGaR shows significant improvement compared to the former, although it still results in a very coarse reconstruction with evident critical issues. As previously mentioned, 3DGS can suffer from inconsistencies due to its viewpoint dependency, which is reflected in the mesh model reconstruction in the form of protrusions and three-dimensional blobs that distort the correct surface. Additionally, there is a complete lack of reconstruction at the base of the bottle.

The most faithful result, which shows impressive outcomes, is based on the 2D Gaussian splatting process. The glass bottle is completely reconstructed and rendered without important heterogeneity. This significant achievement is due to the fact that, compared to 3DGS, 2DGS offers a more robust and reliable solution, maintaining a consistent and precise representation from any angle.

It is worth noting that, compared to the ground truth, the 2DGS mesh appears smoothed and still requires improvement.

Below, the Figure 4 shows the visual comparisons of the meshes reconstructed by the different processes.

### 5.2. Quantitative Analysis

The quantitative analyses were performed through deviation analyses in CloudCompare using the C2M method, with the reference mesh considered as the ground truth. To generate point clouds from the meshes produced in the three parallel processes, a sampling operation was carried out using CloudCompare’s “Points Sampling on Mesh” tool, setting a fixed number of 10 million points for each process. The sampling is carried out proportionally to the surface area of the mesh faces, thus ensuring a uniform geometric representation with an equal number of points for each model.

Furthermore, CloudCompare enables the generation and management of scalar fields associated with point clouds. Each scalar field assigns a numerical value to every point, which can be visually represented through a corresponding color scale. This color mapping translates scalar values into specific colors, facilitating an intuitive and immediate interpretation of spatial variations in parameters such as distances, deviations, or other measured attributes. For instance, regions exhibiting higher deviation values are typically displayed in warmer colors (e.g., red), while lower values are shown in cooler colors (e.g., blue), thereby providing a clear visual overview of the distribution and magnitude of the analyzed metrics.

**Figure 5:**
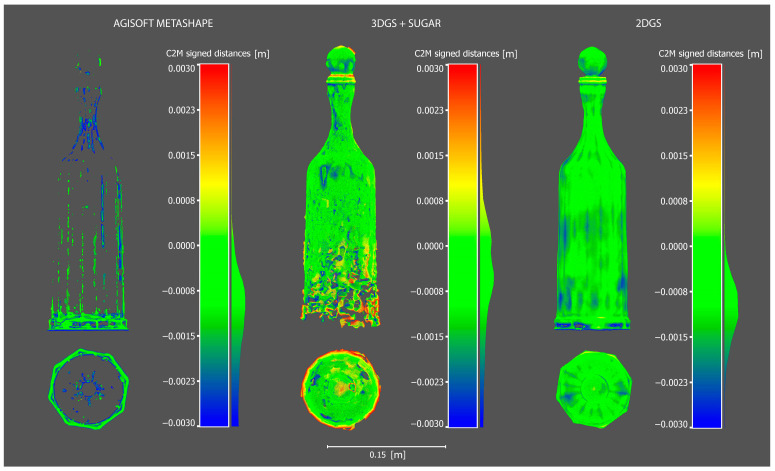
Cloud-to-mesh of the three generated model using as reference the ground truth.

As can be seen from the Figure 5 and Table 4, the C2M deviation analysis once again clearly demonstrates that the most robust reconstruction is the one produced by the 2DGS process. It is worth noting that the data highlighted has been cleaned of out-of-range [−3 mm to +3 mm] points.

#### 5.2.1. Completeness Evaluation of Reconstructed Models

To assess the completeness of the reconstructed models, we compared their surface areas to the ground truth mesh. Completeness (*C*) is computed as in Formula (1):

(1) $$C=\left(\frac{S\;model}{S\;gr\_tr}\right)\times 100$$

where:

- S model is the total surface area of the reconstructed model after out-of-range points removal.
- S gr_tr is the surface area of the ground truth mesh.

The 3DGS model achieved the highest completeness (99.62%), closely followed by 2DGS (96.43%), while AM exhibited significantly lower completeness (16.96%), indicating that it reconstructed only a small portion of the reference surface.

Table 5 summarizes the key attributes of the ground truth mesh and the three reconstructed models (2DGS, AM, and 3DGS). The table includes the number of triangles (both original and after out-of-range removal), surface area, border edges, and perimeter for each model. These parameters provide insights into the overall geometry and completeness of the reconstructions.

Additionally, an analysis of border edges and perimeters provides insight into the distribution of the reconstructed surface. The 3DGS model has the largest perimeter (12.44 m), suggesting potential artifacts or noise at the edges, whereas 2DGS has a more compact structure with a lower perimeter (0.722 m). AM shows a highly fragmented reconstruction with a large number of border edges (4289) and an extensive perimeter (3.158 m).

These findings highlight that completeness alone is not sufficient to assess model quality, as variations in surface distribution and boundary fragmentation must also be considered.

It is also possible to deduce from Table 4, based on the number of points out of range, that the most critical and confusing process is the one using AM.

The 3DGS process tends to “fill” the bottle with three-dimensional Gaussians (ellipsoids), while the process based on 2DGS generates very interesting data, even regarding out-of-range points.

CloudCompare provides a comprehensive set of tools for the generation, visualization, and export of histograms related to scalar fields computed on point clouds or meshes. In this context, a histogram is a graphical representation that shows how scalar values are distributed within the dataset. The X-axis represents the range of scalar values, such as distances, elevation, or deviation metrics, while the Y-axis indicates the frequency or number of points falling within each discrete interval, known as a bin.

One of the main advantages of CloudCompare’s histogram tool is the ability to export the histogram data as a CSV file. This feature enables further quantitative analyses and detailed visualizations using external software such as Excel, Matlab, or Python-based platforms. Through these analyses, researchers can derive important statistical parameters including mean values, standard deviations, and skewness, which support a deeper understanding of the dataset’s characteristics.

Histograms are particularly useful in quality assessment and error analysis. They allow for the identification of anomalous points, known as out-of-range points, which may represent measurement errors, noise, or unexpected but significant geometric features. By examining the shape of the histogram, which can be unimodal, bimodal, or multimodal, analysts can identify the presence of distinct regions or layers within the data. Additionally, histograms facilitate the comparison of scalar field distributions within the same point cloud or between different point clouds, making them essential for benchmarking and validation.

Despite these advantages, it is important to note that CloudCompare’s native histogram functionality offers a relatively simple graphical interface. For complex datasets or more advanced statistical models, it is advisable to export the histogram data and perform further processing using specialized tools. Furthermore, CloudCompare currently does not support automatic histogram generation through command-line operations, so the process remains primarily manual.

**Figure 6:**
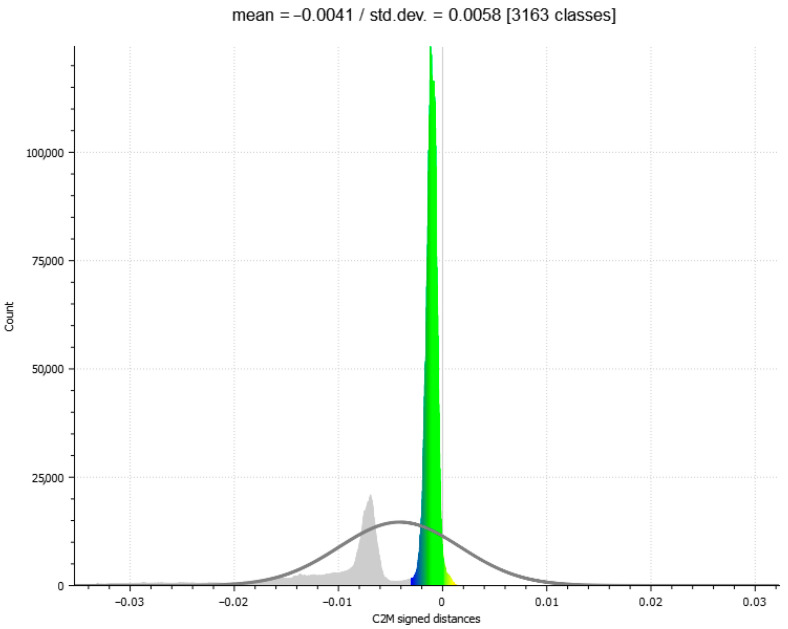
Histogram of the C2M analysis of the 2DGS model considering points out of and in range; a bimodal distribution curve is observed.

**Figure 7:**
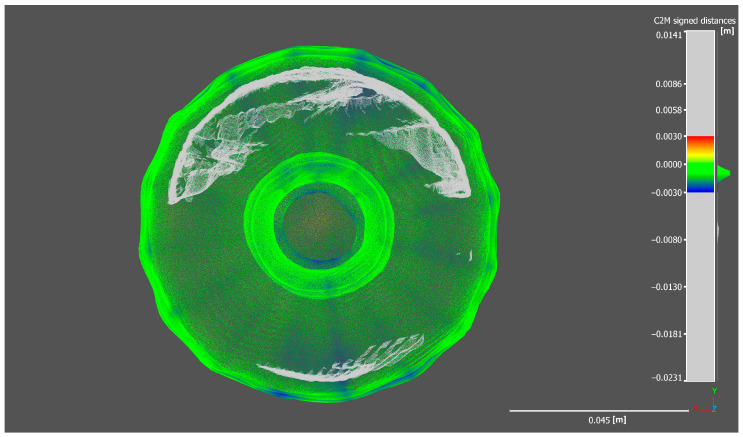
Section of the 2DGS bottle model (bottom view).

**Figure 8:**
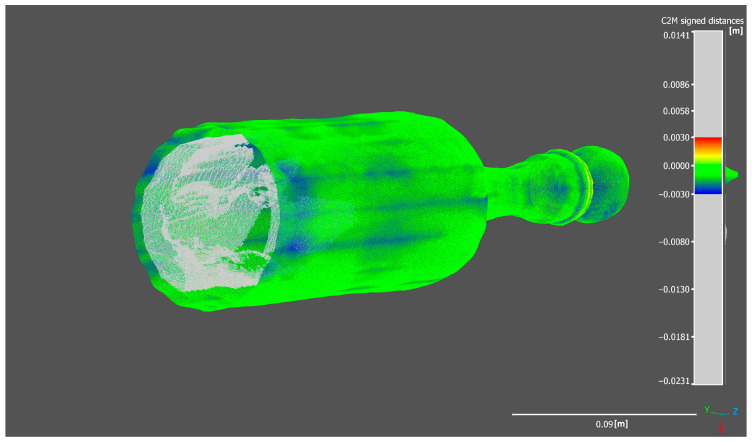
Section of the 2DGS bottle model (axonometric view).

As visible in the exported histogram of the 2DGS model in Figure 6, considering also the out-of-range points, a bimodal distribution curve is observed. Sectioning the bottle while including the out-of-range points reveals the generation of an internal thickness within the bottle (Figure 7 and Figure 8). The points located along this thickness are out of range because the generation of the ground truth does not include the internal surfaces but only the external shell of the bottle.

The histogram shows a sharp and prominent peak near zero, indicating that most points have very small signed distances relative to the reference mesh, which confirms a high level of accuracy in the reconstruction of the external surface. However, the secondary peak, located further away from zero, corresponds to out-of-range points related to the internal structure. These out-of-range points, cause a deviation from the reference model since the internal geometry was not considered during the generation of the reference model.

The Gaussian distribution curve overlaid on the histogram highlights the central tendency and dispersion of the main distribution, with a mean close to zero and a low standard deviation, demonstrating overall precision in capturing the external geometry. The presence of out-of-range points is further confirmed by the spread in the distribution tails, which contributes to the bimodal shape. This analysis emphasizes the importance of accurate interpretation of scalar field histograms, especially when complex geometries with internal features are involved.

Moreover, this bimodal distribution can be useful for filtering or segmenting the point cloud, allowing the separation of points belonging to the external surface from those related to the internal thickness or noise. This can improve the quality of subsequent analyses by excluding points that do not correspond to the modeled surface.

#### 5.2.2. Completeness Evaluation of Reconstructed Models on Slice Sections

In this section, a more detailed analysis is conducted on individual slices of point clouds generated in CloudCompare, allowing for a more precise and practical visualization of how each reconstruction process produces the previously discussed results. Notably, the model generated by the 2DGS process exhibits the highest degree of conformity to the ground truth compared to the other methods. Furthermore, as observed in the previous section, in addition to accurately reconstructing the external surface of the bottle in alignment with the ground truth model, the 2DGS process also captures the internal surface of the bottle, further demonstrating its robustness and completeness.

**Figure 9:**
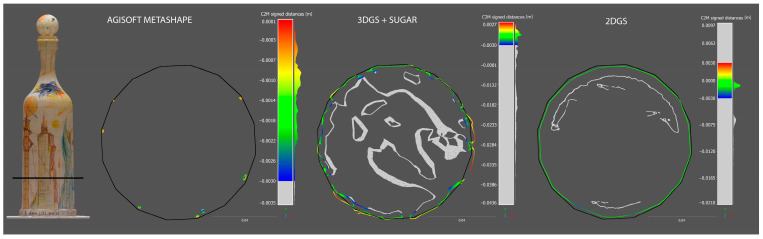
Planimetric C2M slice section of the three generated models using as reference the ground truth (black profile).

**Figure 10:**
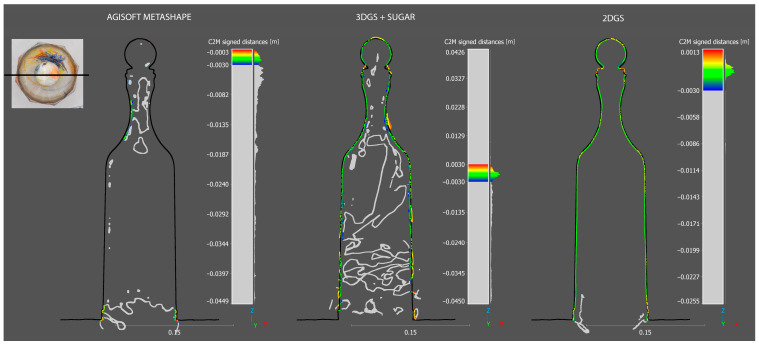
Transversal C2M slice section of the three generated models using as reference the ground truth (black profile).

The models generated by AM remain almost entirely absent. As shown in Figure 9 and Figure 10, the few points within the range are primarily located along the edges of the bottle’s faces.

### 5.3. Rendering Metrics PSNR LLPIS and SSIM for 3DGS and 2DGS

Based on the results obtained from the evaluation of both 3DGS and 2DGS on a challenging transparent glass bottle dataset, we can draw several important conclusions regarding their performance also across the multiple rendering metrics (Table 6).

The SSIM score for 3DGS (0.9768) is slightly higher than that of 2DGS (0.9734), indicating that 3DGS preserves the structural integrity and local details of the reconstructed object better. Although the difference is minimal, a change of 0.003 in SSIM can be noticeable in perceptual terms [8], particularly in the preservation of fine details. This suggests that 3DGS provides a slightly superior visual reconstruction, which is important when dealing with objects where fine details are crucial, such as glass objects with complex reflections and refractions.

Similarly, the PSNR for 3DGS (36.06 dB) is better than that of 2DGS (34.91 dB), which implies a superior signal-to-noise ratio and fewer pixel-wise errors in the reconstruction. A PSNR value above 35 dB is considered excellent in visual quality [8], and while both methods perform well, 3DGS clearly has the edge in terms of numerical fidelity, ensuring fewer artifacts and a more precise reconstruction.

The LPIPS score further highlights the perceptual superiority of 3DGS, with a value of 0.0629 compared to 2DGS’s 0.0696. Since LPIPS is a perceptual metric, this difference is significant. A lower LPIPS value means that 3DGS’s reconstruction is visually closer to the original, making it more faithful in terms of human perception. This difference underlines the advantage of 3DGS when it comes to visual fidelity, particularly when assessing high-precision features like transparency and light interaction with complex materials.

When considering the specific challenges posed by transparency, both methods struggle with the inherent complexities of modeling reflections, refractions, and volumetric effects associated with transparent materials. However, 3DGS’s superior performance across all three rendering metrics suggests that it provides a more robust model for handling the complex optical properties of transparent objects. This is likely due to its better 3D modeling capabilities, which allow it to represent volumetric transparency more accurately and maintain depth consistency in the reconstruction. In contrast, 2DGS, while capable of producing solid meshes, does not inherently account for volumetric depth and transparency, which limits its ability to model such materials with high accuracy.

The choice between 3DGS and 2DGS depends on the specific requirements of the task at hand. If the goal is to achieve the highest quality visual reconstruction, especially for complex materials like transparent glass, 3DGS is the better option. However, for tasks involving solid mesh extraction and multi-view consistency, 2DGS remains a highly effective solution.

### 5.4. Processing Times

**Figure 11:**
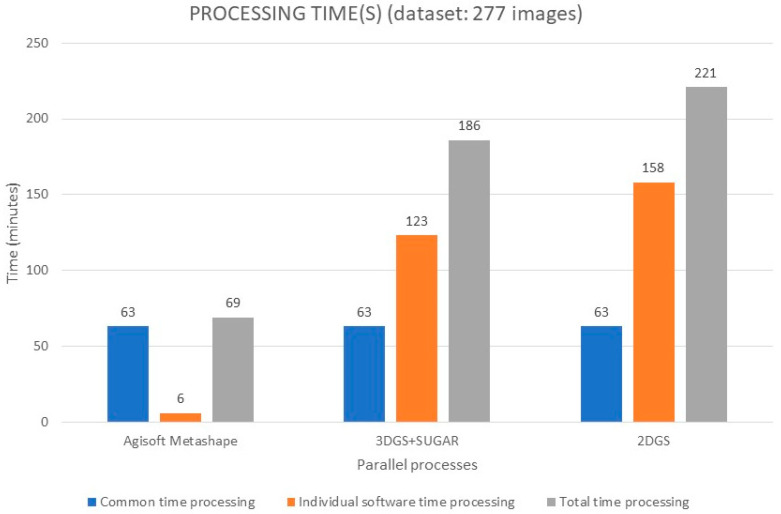
Processing time(s) for each parallel process.

In Figure 11, the processing times for each individual process are shown in orange, while the time common to all three processes accounting for automated masking in Adobe Lightroom and the subsequent alignment of the 277 photographic shots in AM is highlighted in blue. Each test was performed in an isolated environment, ensuring that no other processes or background tasks were running at the same time. This was done specifically to guarantee a fair and consistent comparison of the processing times.

The results highlight that the most time-consuming processes are also the most effective in terms of the geometric reconstruction of the 3D model. Specifically, there is a clear monotonic growth, starting with a very low processing time for AM and reaching a maximum when using the traditional 2DGS method.

#### CPU and GPU Usage

The Table 7 provides an overview of how different computational resources are utilized by 2DGS, 3DGS, and AM. Both 2DGS and 3DGS rely heavily on GPU performance since Gaussian splatting is a highly parallelizable process that benefits from the massive parallel processing power of modern GPUs. The CPU plays only a minimal role in these workflows, primarily handling data preparation and management, while the actual computation is executed on the GPU.

On the other hand, AM takes a more balanced approach, utilizing both the CPU and GPU for different tasks. The CPU is crucial in feature matching, mesh generation, and texturing, which require intensive sequential processing and memory management. However, the GPU plays a vital role in accelerating depth map calculations, point cloud generation, and rendering, significantly reducing processing time.

For users looking to optimize their workflow, it is essential to have a high-performance GPU with extensive parallel processing capabilities, such as an NVIDIA RTX 3090 or 4090, when working with Gaussian splatting techniques. In contrast, AM benefits from both a powerful multi-core CPU and a capable GPU to ensure smooth performance across all computational stages. Understanding these distinctions allows professionals to make informed hardware choices based on their specific computational needs and workflow requirements.

## 6. Discussion

This study proposed a comprehensive comparative analysis of three different 3D reconstruction methodologies AM, 3DGS + SuGaR, and 2DGS, evaluating their performance in terms of completeness, geometric accuracy, and reconstruction consistency, integrating image-based rendering metrics (SSIM, PSNR, LPIPS) with quantitative geometric metrics related to transparent surfaces.

### 6.1. Comparative Performance Analysis

Quantitative and qualitative evaluations highlighted significant differences among the analyzed methodologies. AM showed the lowest reconstruction capability, exhibiting a high level of fragmentation and an extremely limited superficial reconstruction. The resulting model presents large gaps, especially in smooth and transparent areas, such as the bottle surface. This limitation is mainly attributed to the intrinsic difficulties of photogrammetry in handling transparent surfaces: SfM and MVS algorithms rely on feature matching across multiple images to generate point clouds and surfaces, but highly transparent objects like glass pose a significant challenge, as it is difficult to distinguish the actual surface from the environment visible through the object, leading to missing parts or distortions in the reconstruction.

In comparison, the 3DGS + SuGaR method achieves higher completeness (99.62%) and provides an improved reconstruction over AM. However, it presents multi-view inconsistencies, resulting in distortions of the reconstructed surface, evident through the presence of surface artifacts, protrusions, and an extended perimeter (12.44 m), indicating noise and imprecise edge definition.

The 2DGS method emerges as the most effective, offering high completeness (96.43%) while maintaining a compact and accurate surface representation. Unlike 3DGS, 2DGS ensures better consistency among views and produces a more stable and homogeneous geometry. Furthermore, the 2DGS process reconstructs not only the external surface of the bottle but also the internal one, a feature absent in the other methods, demonstrating the technique’s potential in capturing fine details and complex geometric structures.

3DGS stands out for superior visual and numerical rendering of transparent objects, as confirmed by higher scores in SSIM and PSNR and lower scores in LPIPS. This indicates greater suitability of 3DGS for rendering tasks and visual fidelity, where preserving optical details is crucial. However, 2DGS excels in extracting compact and multi-view consistent meshes, suitable for downstream applications such as digital twin, 3D printing, CAD, and simulations. While 3DGS is optimized for realistic rendering, it does not guarantee solid geometric consistency, penalizing the generation of robust geometries; conversely, 2DGS is more suited for tasks requiring solid meshes, even at the expense of some of 3DGS’s visual quality.

### 6.2. Key Findings and Implications

While completeness alone does not determine reconstruction quality, the 2DGS method achieves an optimal balance between high completeness and accurate geometric representation. The 3DGS + SuGaR method, despite its high completeness percentage, produces excessive surface noise, while AM fails to reconstruct a significant portion of the model, particularly in challenging areas.

#### Out-of-Range Points Distribution and Robustness

The error deviation analysis and histogram evaluation highlight how the proposed methodology, which integrates rendering metrics (SSIM, PSNR, LPIPS) with geometric metrics (standard deviation, RMSE, completeness), produces a bimodal error distribution, due to the generation of an internal thickness absent in the reference model. This indicates that, while ensuring a more complete surface representation, alignment with the reference model requires further refinement for applications where the external geometry is the primary focus.

Geometric analyses also highlighted significant aspects: the cross-sectional (slice) analysis of the bottle clearly shows how the 2DGS process can reconstruct some internal surface parts of the object, confirming its effectiveness in capturing complex volumetric and structural details. For a more comprehensive overview of the results, a video demonstrating the overall outcomes has been included in the Supplementary Materials.

## 7. Conclusions, Limitations and Future Works

Although the proposed methodology characterized by the integration of image-based rendering metrics (such as SSIM, PSNR, and LPIPS) and quantitative geometric metrics (such as standard deviation, mean, and completeness) has proven effective in evaluating the accuracy of 3D reconstructions of transparent objects, some limitations must be acknowledged. The validation was conducted on a single dataset consisting of a transparent glass object with relatively simple and regular geometry. While this choice ensured a high level of experimental control and reproducibility, it limits the generalizability of the results.

Future work will focus on applying this integrated evaluation framework to a broader and more heterogeneous set of transparent case studies with also different lighting conditions. It will be particularly important to include objects with greater morphological complexity, preferably belonging to cultural and historical heritage, thereby extending the application scope beyond the industrial and design sectors. This direction will allow for a more rigorous assessment of the methodology’s robustness and address the challenges posed by irregular and historically significant objects.

Moreover, as noted in Section 2.1.2, at the time of this study, several recent and promising reconstruction techniques had not yet been formally published or did not have open-source implementations available. Given the rapid evolution of Gaussian splatting and its applications to transparent surface reconstruction, these methods could not be included in the experimental evaluation. Future research will focus on integrating and benchmarking these emerging techniques as they become accessible, with the aim of further improving precision, reproducibility, and applicability of 3D reconstruction workflows in complex optical scenarios.

Simultaneously, a crucial improvement will involve replacing the alignment phase currently based on proprietary software such as AM with a fully open-source pipeline. This will likely include adopting deep-image-matching [45] techniques, which have demonstrated greater robustness in multi-view feature extraction and matching. Likewise, the use of Adobe Lightroom Classic for image pre-processing (e.g., exposure correction and automatic masking) will be reconsidered in favor of open-source solutions. Transitioning to a fully open and replicable workflow will reduce dependency on proprietary software, promoting transparency, scientific reproducibility, and wider adoption by the research community.

## Figures and Tables

**Table 1:** Common settings of image acquisition.

Camera’s Characteristics			
**Name**	**Image dimension**	**Focal lenght**	**Sensor dimensions**
Nikon D750	6016 × 4016 pixels	50 mm	W = 36.0 mm H = 23.9 mm
**Common Settings of Image Acquisition**			
**Aperture**	**Shutter speed range (Aperture priority mode)**	**ISO**	**Format**
f/16	1/8–1/10	200	RAW

**Table 2:** Common alignment parameters.

Accuracy	Limit Key Points	Limit Tie Points	Generic Preselection	Reference Preselection	Adaptive Camera Model Fitting	Exclude Stationary Tie Points	Guided Image Matching
High	0	0	No	No	No	Yes	No

**Table 3:** Agisoft Metashape parameters to generate mesh model.

Source Data	Surface Type	Quality	Face Count	Interpolation	Depth Filtering
Depth Maps	Arbitrary	High	High	Enabled	Mild

**Table 4:** Quantitative parameters extracted from the C2M analysis.

Processes	Mean	St. Deviation	RMSE	Points in Range	Points out of Range
Agisoft Metashape	−0.0014	0.0008	0.00161	2,889,373	7,110,741
3DGS + SUGAR	−0.0006	0.0011	0.00125	3,894,545	6,105,469
2DGS	−0.0011	0.0005	0.00121	6,870,297	3,129,632

**Table 5:** Key attributes of the ground truth mesh and the three reconstructed models (2DGS, Agisoft Metashape, and 3DGS).

Model	Completeness	Triangles (Original)	Triangles (After Out-of-Range Removal)	Surface Area (m^2^)	Border Edges	Perimeter (m)
Ground Truth	-	255,971	-	0.080029	425	0.370151
Agisoft Metashape	16.96%	188,267	76,137	0.013567	4289	3.158630
3DGS + SUGAR	99.62%	69,979	33,096	0.079725	5146	12.443651
2DGS	96.43%	206,201	141,498	0.077172	642	0.722261

**Table 6:** Rendering metrics PSNR LLPIS and SSIM (visual fidelity) for 3DGS and 2DGS compared with the mesh accuracy.

Method	SSIM	PSNR	LPIPS	Visual Fidelity	Mesh Accuracy
3DGS	0.9768	36.06	0.0629	Higher (more realistic)	Low degree of conformity
2DGS	0.9734	34.91	0.0696	Good, slightly worse	High degree of conformity

**Table 7:** Overview of the different computational resources utilized by 2DGS, 3DGS, and Agisoft Metashape.

Software/Process	CPU Usage	GPU Usage	Notes
Agisoft Metashape	Yes	Yes	CPU used for feature matching, mesh generation, and texturing. GPU accelerates depth maps, point cloud, and rendering.
3DGS + SUGAR	No	Yes	Intensive GPU computation for 3D Gaussian management. CPU marginally used for coordination.
2DGS	No	Yes	Uses GPU for rasterization and Gaussian optimization. CPU involved only in data management.

## Data Availability

The data that support the findings of this study are available from the corresponding author upon reasonable request.

## Supplementary Materials

A video of the overall study is available at https://www.youtube.com/watch?v=d0C4YqTi1xg (accessed on 15 April 2025). The video also demonstrates the access to the SIBR VIEWER for 3D Gaussian Splatting via the Anaconda Prompt.

## Abbreviations

The following abbreviations are used in this manuscript:

SfM	Structure from Motion
MVS	Multi View Stereo
3DGS	3D Gaussian Splatting
2DGS	2D Gaussian Splatting
CH	Cultural Heritage
AI	Artificial Intelligence
CV	Computer Vision
NeRF	Neural Radiance Field
MLP	Multi-Layer Perceptron
CNN	Convolutional Neural Network
LPIPS	Learned Perceptual Image Patch Similarity
PSNR	Peak Signal-to-Noise Ratio
SSIM	Structural Similarity Index Measure
SuGaR	Surface-Aligned Gaussian Splatting for Efficient 3D Mesh Reconstruction and High-Quality Mesh Rendering
GS2Mesh	Gaussian Splatting-to-Mesh
GOF	Gaussian Opacity Fields
MVG-Splatting	Multi-View Guided Gaussian Splatting
AM	Agisoft Metashape
RMSE	Root Mean Square Error
GPU	Graphics Processing Unit
CPU	Central Processing Unit
